# Diversification of tobacco phytoene-synthase-encoding genes coordinates carotenoid flux, strigolactone biosynthesis, and cold responses

**DOI:** 10.3389/fpls.2026.1750572

**Published:** 2026-04-10

**Authors:** Xiaoxu Li, Wei Luo, Suxing Tuo, Long Chen, Jiaruo Huang, Ye Feng, Xiangguang Lv, Jinghao Sun, Guoyun Xu, Jun Cai, Jianfeng Zhang, Zhiyuan Li, Bo Kong

**Affiliations:** 1 Beijing Life Science Academy, Beijing, China; 2 Tobacco Chemistry Research Institute of Technology Center, China Tobacco Hunan Industrial Co., Ltd., Changsha, China; 3 Tobacco Research Institute, Chinese Academy of Agricultural Sciences, Qingdao, China; 4 Zhengzhou Tobacco Research Institute of China National Tobacco Corporation (CNTC), Zhengzhou, China; 5 Hunan Key Laboratory of Plant Functional Genomics and Developmental Regulation, Hunan Research Center of the Basic Discipline for Cell Signaling, College of Biology, Hunan University, Changsha, China

**Keywords:** metabolism, *NtPSY1*, phytoene synthase, stress tolerance, tobacco

## Abstract

Phytoene synthase plays important roles in the development, metabolism, and various stress tolerance in plants. In the current study, a total of five tobacco phytoene synthase members were identified and systemically analyzed from the latest genome annotation. The newly identified tobacco members were divided into different subgroups together with the reported members. Furthermore, four *NtPSY* genes were identified to arise from duplication events, which might lead to the expansion of tobacco phytoene synthase members. The expression patterns of the *NtPSY* genes were revealed by transcriptomic analysis. The results from phylogenetic analysis, synthetic analysis, and expression analysis were integrated to predict the potential functions of these tobacco phytoene synthase members. Particularly, *NtPSY1* was found to act as the homolog of *SlPSY1*, which was significantly induced by cold stress treatment. The further assays revealed that overexpression of *NtPSY1* enhanced the carotenoid accumulation and cold stress tolerance in transgenic tobacco. Furthermore, the transcript levels of *NtCCD7*, *NtCCD8*, and *NtMAX1* were detected to be increased in the *NtPSY1* overexpression lines, suggesting the enhanced carotenoid precursor supply and activation of the strigolactone biosynthetic pathway.

## 1 Introduction

In the plant kingdom, individuals inhabit fluctuating environments and continually face developmental and stress challenges. The carotenoid pathway provides indispensable photoprotective pigments and precursors of apocarotenoid hormones, and its first committed step, often rate-limiting, is catalyzed by phytoene synthase (PSY) (Cao et al., 2019; Zhou et al., 2022). PSY typically occurs as a small multigene family in plants, with isoforms exhibiting distinct spatiotemporal expression and regulatory behaviors to tune carotenoid flux during growth, morphogenesis, and stress acclimation (Zhou et al., 2022).

Across angiosperms, PSY diversification underpins functional specialization. In maize (*Zea mays*), three PSY paralogs partition functions across tissues and environments. *ZmPSY1* is required for endosperm carotenoid accumulation and underlies natural grain color variation (Fu et al., 2013). *ZmPSY2* primarily supports photosynthetic tissues, and *ZmPSY3* is root-biased and stress-inducible (drought/salt/ABA), linking carotenoid flux to stress physiology and rhizosphere signaling (Buckner et al., 1996; Li et al., 2008a, Li et al., 2008b). Consistent with these roles, PSY1 is a major contributor to quantitative variation in kernel provitamin A, and combinatorial overexpression strategies identified PSY as a strong leverage point for biofortification (Zhu et al., 2008; Diepenbrock et al., 2021). In rice (*Oryza sativa*), *OsPSY1*/*OsPSY2* act predominantly in photosynthetic tissues, whereas *OsPSY3* constitutes a stress-responsive root isoform that is induced by salt and drought and coordinates with ABA biosynthesis to sustain carotenoid-derived hormone production under stress (Welsch et al., 2008). The centrality of PSY to flux control is further underscored by Golden Rice, in which the endosperm expression of *ZmPSY1* enabled high β-carotene accumulation and proved bioavailable in humans (Paine et al., 2005; Tang et al., 2009). In *Solanaceae*, tomato exemplifies the canonical triad (*PSY1*/*PSY2*/*PSY3*) with partially overlapping yet specialized roles in fruit chromoplasts, green tissues, and roots, respectively (Cao et al., 2019). In parallel, PSY activity integrates with upstream GGPP supply and protein partners to allocate isoprenoid flux under environmental cues (Zhou et al., 2022). Moreover, PSY family members can also be stress-responsive—for example, in potato, *StPSY1–3* are induced by cold, highlighting potential roles for PSY in abiotic stress tolerance (Kulakova et al., 2024).

Notably, an ancestral, symbiosis-regulated clade of *PSY* genes determines root strigolactone (SL) levels, coupling PSY-driven carotenoid flux to rhizosphere signaling and arbuscular mycorrhizal symbiosis (Stauder et al., 2018). This discovery reframes PSY as a nexus between primary carotenoid biosynthesis and SL-dependent developmental and ecological processes, with obvious implications for root biology and nutrient foraging.

Tobacco (*Nicotiana tabacum* L.) is both an economically important crop and a tractable *Solanaceae* model. Leveraging reference tobacco genomes and transcriptomes enables the systematic discovery of carotenoid pathway genes and isoforms at the family scale. Building on this genomic foundation, a prior work identified six PSY coding sequences in tobacco (organized previously as *PSY1-1*/*PSY1-2*, *PSY2-1*/*PSY2-2*, and *PSY3-1*/*PSY3-2*), consistent with its allotetraploid origin from *N. sylvestris* and *N. tomentosiformis*. *PSY1*/*PSY2* predominate in aerial tissues, whereas *PSY3* shows a limited or condition-dependent expression (Wang et al., 2021). With curated genomic resources now available for tobacco, a comprehensive *in silico* to experimental survey of the tobacco PSY family is both feasible and timely.

In this study, we perform a genome-wide identification and comparative analysis of the *PSY* gene family in tobacco, evaluate their phylogenetic relationships within *Solanaceae*, characterize conserved motifs and gene structures, and profile expression across tissues and stress treatments. In this case, we further explore how the tobacco PSY diversification intersects with carotenoid-derived hormones, particularly the SL pathway spotlighted by the symbiosis-regulated PSY clade, to inform targets for metabolic engineering of photosynthetic performance, stress resilience, and quality traits.

## 2 Materials and methods

### 2.1 Identification and classification analysis of tobacco PSY members

The genome sequence and annotation information of tobacco was downloaded from the Nicomics database (http://lifenglab.hzau.edu.cn/Nicomics). The previously reported *AtPSY* and *SlPSY* full-length protein sequences (Cao et al., 2019) were obtained from The *Arabidopsis* Information Resource (TAIR) and the SGN database and used as queries to perform the BLASTP search against the annotated tobacco protein databases with an *E*-value cutoff of 0.01. Furthermore, the HMM search was performed against the annotated tobacco protein databases under the *E*-value cutoff of 0.001. The candidate sequences from the two above-described approaches were integrated, and redundant entries were removed manually. The putative PSY sequences were analyzed with both Pfam (https://pfam.xfam.org/) with an *E*-value cutoff of 1.0 and SMART (http://smart.embl.de/) with an *E*-value cutoff of 1.0 to detect the presence relative domain.

The full-length protein sequences of tomato PSY members and newly identified tobacco PSY members were subjected to multiple sequence alignment with MAFFT v5.3 under the default settings. The alignment of the PSY domain was visualized by using TBtools (Chen et al., 2023). Subsequently, a neighbor-joining phylogenetic analysis was conducted by using MAGE X based on the alignment of full-length protein sequences with the bootstrap method of 1,000 replicates, substitution with Poisson model, and pairwise deletion (Kumar et al., 2018). The phylogenetic tree was displayed with FigTree v1.3.

### 2.2 Exon–intron structural analysis and identification of conserved motifs

The genomic sequences and coding sequences of *SlPSY* and *NtPSY* members were submitted to the Gene Structure Display Server to visualize their exon–intron structure (Hu et al., 2015).

### 2.3 Chromosomal localization and duplication event analysis

According to the location information provided by the *Solanaceae* database, the Perl program was adopted to display the *NtPSY* members’ chromosomal location. Afterward, the TBtools’ Circos program was recruited to analyze the synteny relationship of the orthologous genes from tobacco and other species.

### 2.4 Promoter analysis of tobacco *PSY* genes

The sequences 2,000 bp upstream of the *PSY* genes in tobacco were extracted from the genome sequence database. The obtained sequences were subjected to PlantCARE platform analysis to further search for the putative *cis*-elements in their promoter regions (Lescot et al., 2002).

### 2.5 Analysis of expression patterns

The reported RNA-seq data of tobacco tissues (Sierro et al., 2014) were downloaded from the GEO database (accession number: PRJNA208209). The RNA-seq data of the tobacco cold treatment were produced in our previous work (Zhou et al., 2018) and were uploaded at the NCBI Short Read Archive (SRA) under the accession number SRP129465. The processed expression data of the *PSY* genes were extracted (**Supplementary Table 1**) and normalized by using TBtools under the default parameters.

### 2.6 Preparation and stress treatments of tobacco plants

Cultivated tobacco K326 was used to analyze the expression pattern of *NtPSY* in this study. Transgenic and wild-type (K326) tobacco seeds were sterilized and grown in vertical MS medium for 14 days and transferred to the soil for 30 days. For the cold stress treatment, these lines were treated at 10°C for 2 weeks; then, the tissues were collected and frozen in liquid nitrogen. The samples were stored in the refrigerator at -80°C for later use.

### 2.7 RNA extraction and qTR-PCR

Total RNAs from each sample were extracted by using Ultrapure RNA Kit (cwbiotech, Beijing, China). Then, the first-strand complementary DNA (cDNA) was synthesized using Evo M-MLV Mix Kit with gDNA Clean for qPCR (Accurate Biotechnology, Changsha, China). The quantitative real-time PCR (qRT-PCR) reactions were performed in the Roche Light Cycler 480 Real-Time PCR instrument with SYBR^®^ Green Premix Pro Taq HS qPCR Kit (Accurate Biotechnology, Changsha, China). The tobacco ribosomal protein gene *L25* (GenBank no. L18908) was used as the control (Li et al., 2021). All experimental data were obtained through three technical repetitions and three biological replicates, and the relative expression level was calculated by using the 2^-△△CT^ method. Details of the primers are provided in **Supplementary Table 1**.

### 2.8 Tobacco transgenic plant and sample preparation

The coding sequence of the *NtPSY1* gene was amplified from the cDNA and inserted into the pCHF3 vector, which was driven by the *CaMV-35S* promoter, to complete the construction of the overexpression vector. The pCHF3 plasmid containing the *NtPSY1* gene was transformed into tobacco leaves by the *Agrobacterium*-mediated method. T0 generation seeds were screened by using MS medium with 50 mg/L kanamycin to obtain T1-generation positive seedlings. T3-generation seeds and wild-type (K326) seeds were sterilized and grown in vertical MS medium for 14 days and then transferred to the soil for 30 days. For the cold stress treatment, these lines were treated at 10°C for 2 weeks and then the survival rates were measured, with nine plants per group and a total of three groups for biological replicates. For flue-cured leaves, the middle leaves were collected from tobacco plants after transplantation for 70 days, and the leaves were cured as described previously (Li et al., 2017). The β−carotene content was determined using a commercial plant beta carotene ELISA kit (MyBioSource, MBS9373360) according to the manufacturer’s instructions. Each treatment contains five cured middle leaves, and three biological replicates were conducted for each treatment. The significant difference analysis was calculated by using SPSS v18.0 with the *t*-test.

## 3 Results

### 3.1 A total of five PSY members were identified in the latest tobacco genome

To catalogue phytoene-synthase-encoding genes in *Nicotiana tabacum*, we performed BLASTP- and HMMER-based homology searches using *Arabidopsis* and tomato PSY proteins as queries. These analyses retrieved five putative *PSY* genes from the most recent tobacco reference genome. The *NtPSY* genes are nonuniformly distributed across chromosomes. For clarity, newly identified genes were designated according to their physical order along the chromosome. Full annotations are provided in **Supplementary Table 2**.

### 3.2 The tested PSY members were grouped by phylogenetic analysis

**Figure 1:**
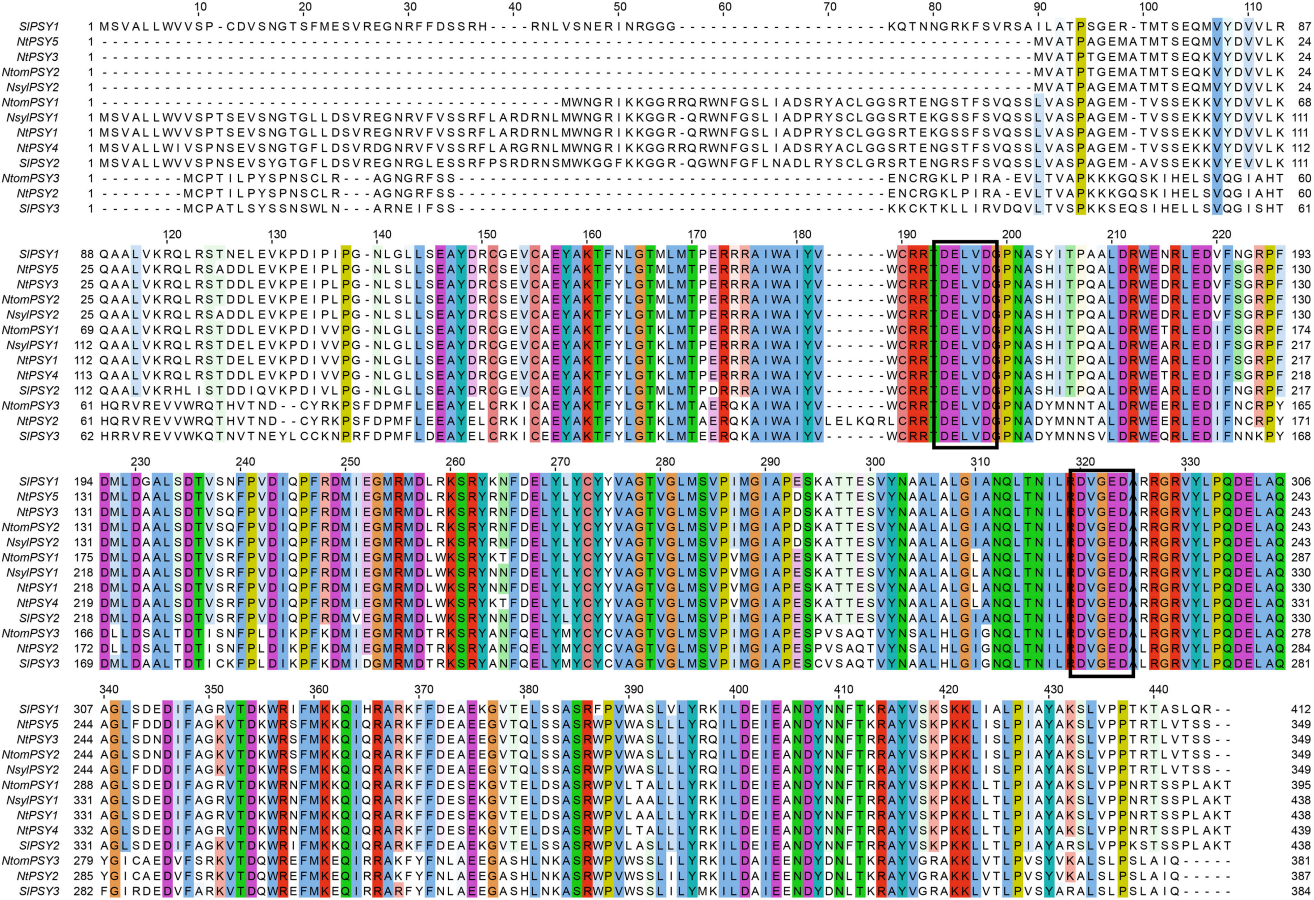
Phytoene synthase active site motif analysis of tobacco and typical PSY members.

The five tobacco PSY homologs encode proteins of comparable lengths and share a conserved phytoene synthase active site motif (DXXXD) containing four invariant aspartate residues (**Figure 1**). The protein sequence alignment revealed a high degree of identity among the homologs, underscoring the high conservation of the core catalytic domains.

**Figure 2:**
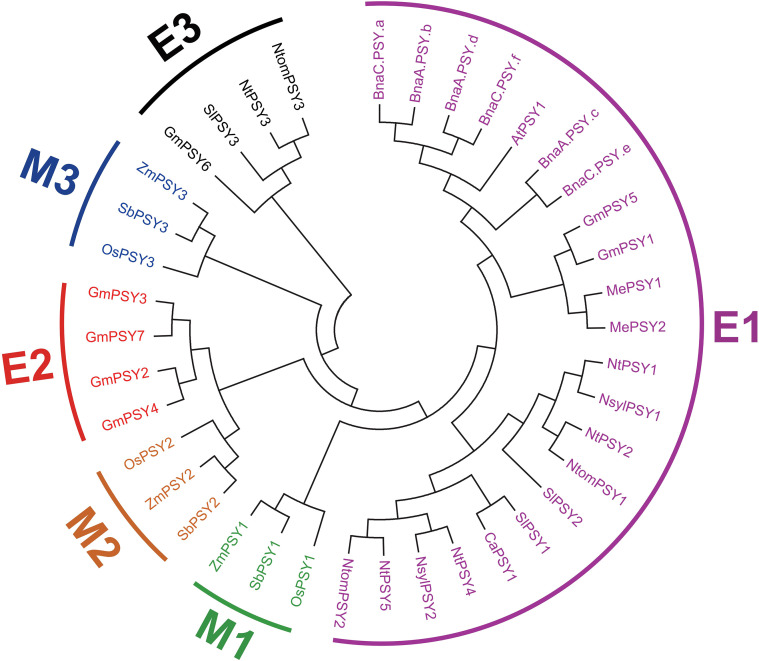
Phylogenetic analysis of tobacco and typical PSY members. The tobacco PSY members together with their *Arabidopsis* and other homologs were classified into six subgroups.

As the result, all of the PSY members were divided into six subgroups (**Figure 2**). Among them, E1 to E3 subgroups harbored the members from dicots, while M1 to M3 contained members from monocots. As the result shows, none of the subgroups had members from those two classifications, indicating that the expansion of PSY members may appear before the divergence of monocots and dicots. Interestingly, subgroup E1 contained more PSY members from tobacco species than *Arabidopsis* and tomato, implying that duplication of events might have occurred in this subgroup. Notably, it was found that subgroup E3 only harbored one PSY member from tobacco, whereas two members, namely, NtPSY3–1 and NtPSY3-2, were reported in a previous study (Wang et al., 2021).

### 3.3 The identified PSY within the same group share a similar gene structure

A comparative analysis of gene structure, particularly intron–exon organization, can provide valuable insights into the evolutionary history of gene families. The exon–intron structures of the five *NtPSY* genes were examined and found to vary considerably, with exon numbers ranging from five to six (**Supplementary Figure 1**). The *NtPSY* genes shared similar gene structures with the tomato *PSY* genes in the same group. Among them, *NtPSY1*, *NtPSY2*, *NtPSY3*, and *NtPSY4* each contain six exons. Notably, *NtPSY5* exhibits a distinct gene structure comprising five exons.

### 3.4 The *NtPSY* homologs were authenticated by syntenic analysis

**Figure 3:**
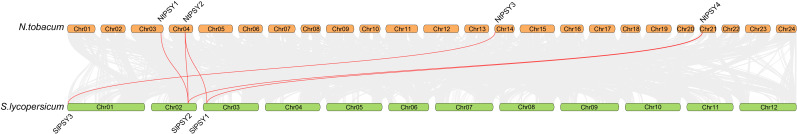
Synteny analysis of *PSY* genes between tobacco and tomato. The gray line in the background represents the collinear blocks between tobacco and tomato, while the collinear *PSY* gene pairs are highlighted by the red color.

Syntenic analysis is important in genome sequence comparisons, which reveal genomic the evolution of different species, while syntenic pairs are predicted as orthologs and might share similar functions (Wang et al., 2021). As a result, four collinearity pairs of PSY members were found in tobacco and tomato (**Figure 3**). The collinear pairings between four of the *NtPSY* genes with PSY members in tomato were identified. Furthermore, no tobacco *NtPSY* genes were identified to form collinear pairs with *PSY* genes from all of the tested monocots species, including rice and maize, implying that no *PSY* genes may have existed before the divergence of these species. All of the *PSY* collinear pairs were predicted between tobacco and tomato but not found in the tested monocotyledonous plants, suggesting that these pairs might arise after the divergence of dicotyledonous and monocotyledonous plants. The details of the tobacco *PSY* syntenic pairs can be found in **Supplementary Table 3**.

### 3.5 Segmental duplication events played a major role in the expansion of *NtPSY*

**Figure 4:**
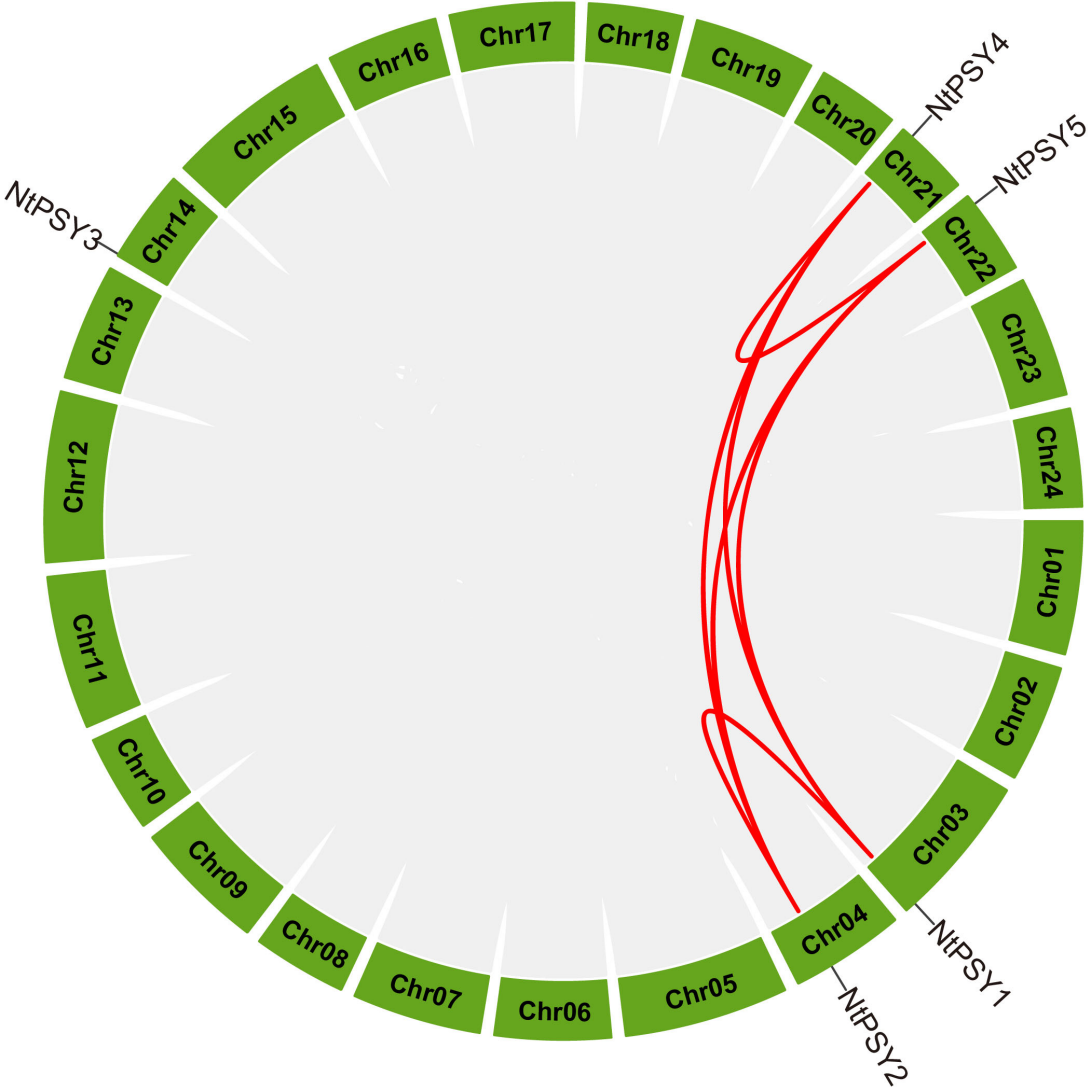
Distribution of tobacco *PSY* genes on chromosomes and segmental duplication events. All of five tobacco *PSY* genes anchored on tobacco chromosomes. The segmental duplication pairs of *NtPSY* genes were predicted by MCScanX and linked by the red lines, respectively. In addition, the gray lines stand for all putative segmental duplication pairs in the tobacco genome sequences.

The chromosomal location information of five *NtPSY* genes was obtained from the latest tobacco genome resource and visualized using R. As a result, chromosomes 03/04/14/21/22 were found to hold one *NtPSY* gene (**Figure 4**). The gene segmental duplication event served as an important access for plants to acquire new genes and gene family expansion. As a result, a total of four tobacco *PSY* genes were identified to form segmental duplication pairs. Notably, these results suggested that about 50% of the *NtPSY* genes may be generated by duplication events, which played a major role in the expansion of the *PSY* gene family in tobacco (**Figure 4**).

### 3.6 Abundant developmental and stress responses of *cis*-elements were predicted

**Figure 5:**
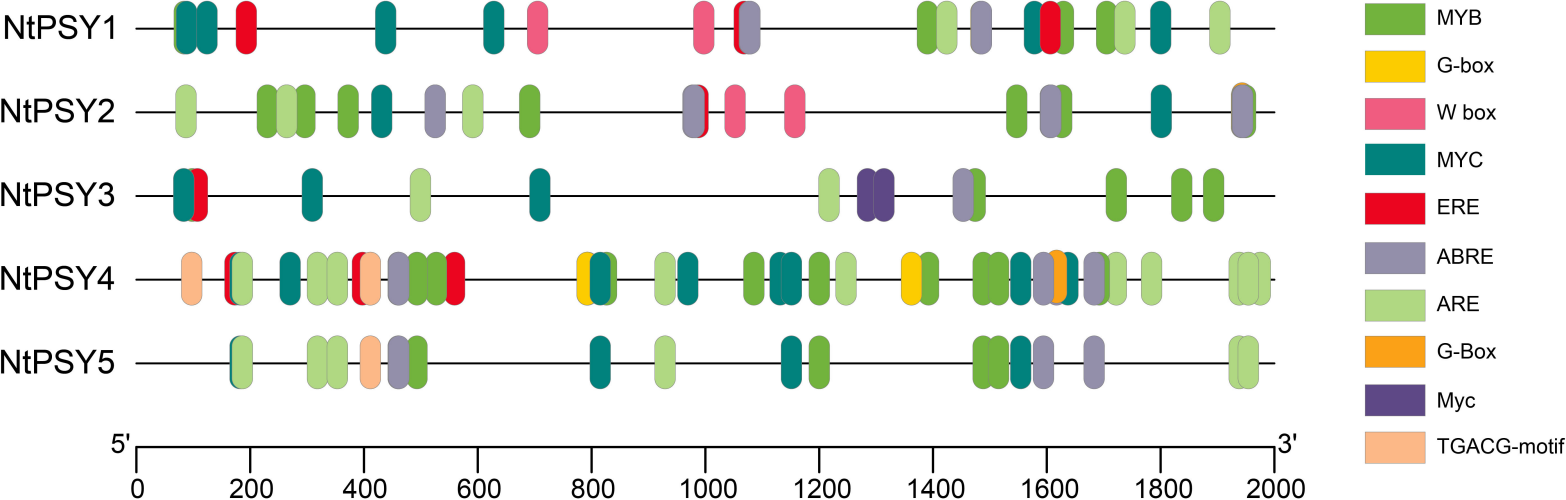
The regulatory *cis*-elements in the promoter regions of tobacco *PSY* genes were predicted by PlantCARE. The color represents different *cis*-elements contained in the promoter sequence.

In a previous study, the *PSY* genes had been reported to be involved in various developmental and stress responses. Considering these clues, the *cis*-element analysis was investigated to explore the probability of these *NtPSY* genes in developmental and stress responses. To study the expression regulation of the *NtPSY* genes, the promoter region of five *NtPSY* genes was analyzed by using PlantCARE Online toolbox. Generally, it was found that a lot of *cis*-elements involved in various developmental and stress responses were present in these tobacco *PSY* gene promoters. Furthermore, 10 *cis*-elements were selected from the PlantCARE database for visualization (**Figure 5**). The results show that all of the identified *NtPSY* gene promoters contained ABRE (abscisic acid responsive *cis*-element), suggesting that those *PSY* genes may function in the abscisic acid signal pathway. Besides that, the *NtPSY1–4* gene promoters harbored ERE (ethylene responsive *cis*-element), while the *NtPSY4* and *NtPSY5* gene promoters possessed salicylic-acid-responsive *cis*-element (TCA element). In addition, the TGACG motif is related to MeJA responsiveness. The *NtPSY4* and *NtPSY5* gene promoters were detected to possess these two kinds of *cis*-element, respectively. The PSY family members were reported to be induced by stress treatments, such as cold stress. The stress-response-related *cis*-elements of tobacco *PSY* gene promoters were also analyzed consequently. The stress-responsive *cis*-elements including MYB (MYB binding site), W box (WRKY binding site), and ARE (anaerobic induction element) were found to be abundant in the promoter regions of many *NtPSY* gene promoters. Interestingly, a total of four *NtPSY* gene promoters were predicted to hold W-box *cis*-elements, which act as the binding sites of WRKY transcription factor, implying that these *NtPSY* genes might be regulated by a certain WRKY transcription factor. Notably, *NtPSY5* seems to hold a less-tested *cis*-element in the promoter region compared with other members. Overall, the promoters of *NtPSY* genes possess abundant *cis*-elements, suggesting that the expression of these *NtPSY* genes might be regulated by multiple factors.

### 3.7 Expression profiles of *NtPSY* in tissues and under cold treatment

**Figure 6:**
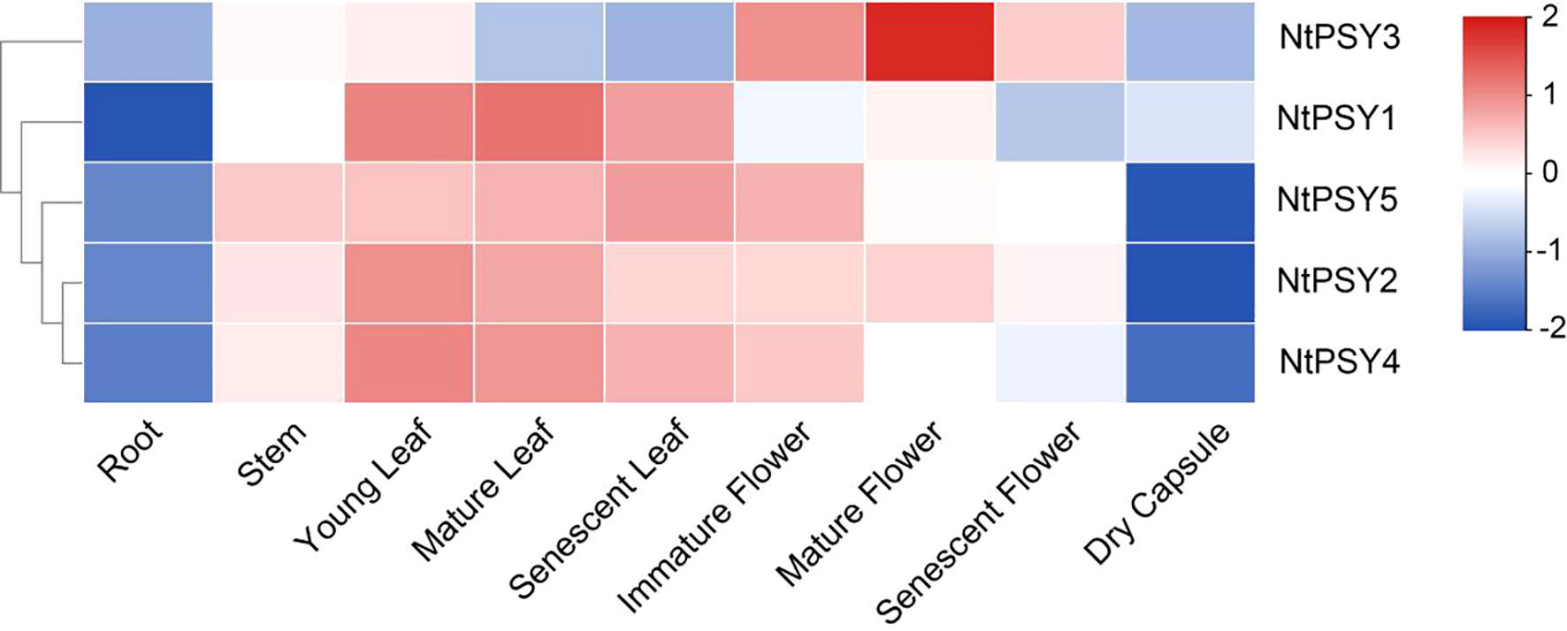
The expression profiles of the *NtPSY* genes in nine different tissues were examined by using transcriptome data. The expression pattern data were retrieved from transcriptome data and visualized by using TBtools under the default parameters.

To preliminarily elucidate the potential roles of *NtPSY* genes in tobacco growth and development, RNA-seq data covering nine distinct tissues were retrieved from the NCBI database and analyzed (**Figure 6**). The results indicated a consistently low transcript abundance of all *NtPSY* genes in root and dry capsule tissues. In contrast, *NtPSY1*, *NtPSY2*, *NtPSY4*, and *NtPSY5* exhibited relatively high expression levels in leaf tissues. Interestingly, *NtPSY3* displayed a distinct expression pattern compared with the other *NtPSY* homologs, with pronounced transcript accumulation observed specifically in mature flowers.

**Figure 7:**
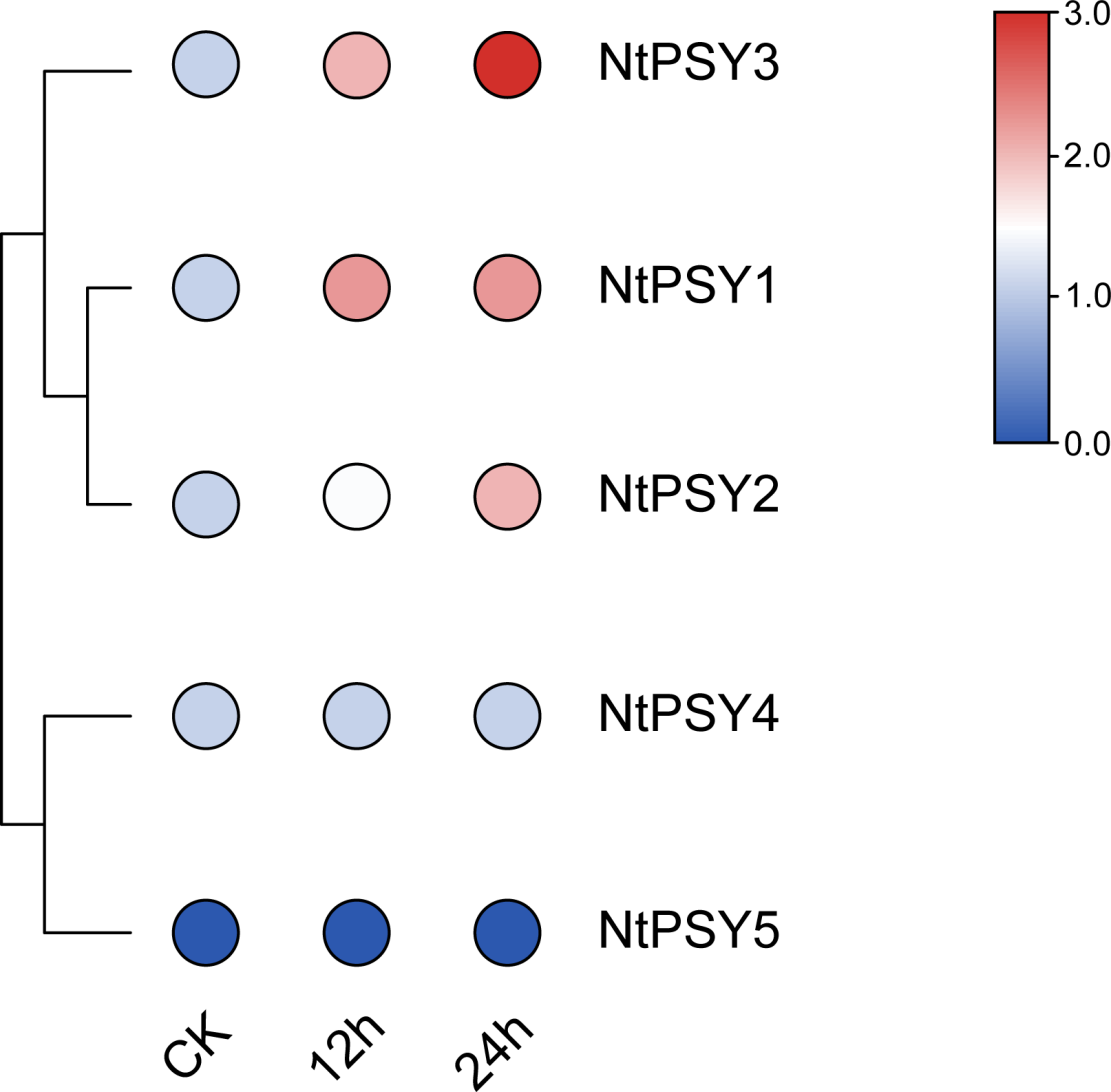
The expression profiles of the *NtPSY* genes under cold treatment were examined by using transcriptome data. The relative expression was visualized by using TBtools under the default parameters. The red, white, and blue colors represent the *NtPSY* expression levels from high to low, respectively.

In addition, another transcriptome analysis revealed expression patterns among the *NtPSY* gene family members in response to cold treatment (**Figure 7**, **Supplementary Table 4**). *NtPSY1*, *NtPSY2*, and *NtPSY3* are induced by cold stress, specifically *NtPSY3. NtPSY3* is highly upregulated, showing a significant increase at 12 h and rising to over six times the control level by 24 h, making it the most responsive to cold. *NtPSY4* remains unchanged, and *NtPSY5* shows no expression in any sample. This indicates that cold stress selectively modulates *NtPSY* genes, notably upregulating *NtPSY1*, *NtPSY2*, and *NtPSY3*.

### 3.8 Richness of expression patterns by qRT-PCR

Furthermore, a number of PSY transcription factors were reported to respond to abiotic stresses in *Solanaceae*. Representative *NtPSY* genes were selected to test whether they could respond to cold stresses. As per the results (**Supplementary Figure 2**), *NtPSY1*, *NtPSY2*, and *NtPSY3* were significantly induced by cold treatments.

### 3.9 NtPSY1 functions in carotenoid flux and cold stress tolerance

**Figure 8:**
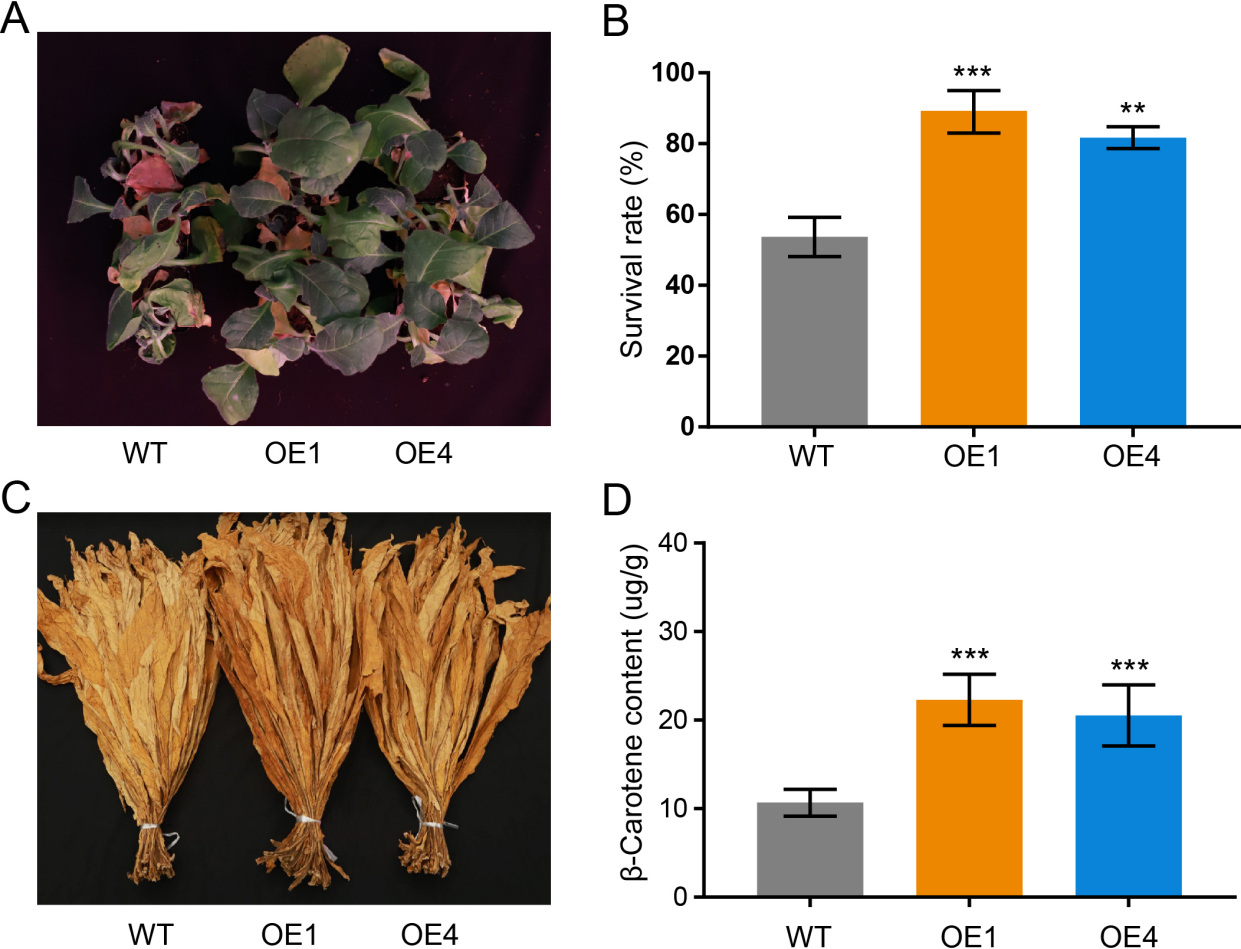
The *NtPSY1* functions in conferring carotenoid flux and cold tolerance. **(A)** Phenotypes of overexpression lines and wild type under cold stress. **(B)** Survival rates of the overexpression lines and wild type, with nine plants per group and a total of three groups for biological replicates. **(C)** Phenotype of flue-cured leaves of overexpression lines and wild type; each sample consists of 30 cured middle leaves. **(D)** β-carotene content of overexpression lines and wild type. Each treatment contains five cured middle leaves, and three biological replicates were conducted for each treatment. The values represent means ± SD. **p < 0.01, ***p < 0.001 (*t*-tests).

Furthermore, the function of *NtPSY1* gene was further examined through genetic experiments. In this study, we generated two stably transformed *PSY1* overexpressed (OE) transgenic plants, with wild type (WT) as control (**Supplementary Figure 3**). Considering that this gene could be induced by cold stresses, the overexpression lines and wild type were treated by cold stress subsequently. After the cold treatment, the overexpression lines and wild-type seedlings displayed a leaf wilting phenotype, whereas the wild-type seedlings were much more extreme than those of the overexpression lines (**Figure 8A**), and the survival rates of the OE-1 lines and OE-4 lines were significantly higher than those of the wild type (**Figure 8B**). The transgenic plants’ flue-cured leaves displayed a deep yellow leaf color phenotype (**Figure 8C**), and the β-carotene content of the overexpression lines were higher than that of the wild type (**Figure 8D**). Hence, the overexpression of the *NtPSY1* gene could improve carotenoid accumulation and cold tolerance in transgenic tobacco.

## 4 Discussion

Phytoene synthase plays important roles in plant development, metabolism, and response to environmental stress. To date, many PSY members have been identified in the genomes of various plant species, such as *Arabidopsis*, rice, and tomato. However, the PSY members in tobacco are not well characterized. In this study, the newly identified tobacco PSY members were studied through a series of analyses. In addition, the *PSY* genes homologous between tomato and tobacco were well studied to investigate their potential functions.

A total of five PSY members were identified from the latest tobacco genome sequences with typical phytoene synthase active site motif (**Figure 1**). Compared with other plant species, tobacco exhibits an apparent expansion of the PSY gene family, with a greater number of PSY members identified than in *Arabidopsis*, wheat, maize, and rice. Gene duplication events have been proven to play important roles in the expansion of gene families (Liu et al., 2026). In our study, syntenic analysis could visualize the location of the homologous or orthologous genes, and the presence of collinear *PSY* genes in different species may have conserved functions, which gives an insight into the functions of the PSY members. In the current study, we identified the collinear pairs of the *PSY* genes with tomato. A total of four tobacco *PSY* genes were identified to form collinear pairs with tomato (**Figure 3**), albeit those four collinear *PSY* genes were distributed in different subgroups (**Figure 2**), suggesting that these *PSY* genes may have existed before the divergence of these two species. Gene duplication has played a very important role in the expansion of gene families. In the current study, a total of four duplication events were identified, all of which involved segmental duplication (**Figure 4**). This discovery implied that the duplication events might play an important role in the evolution of the tobacco *PSY* gene family.

Furthermore, only one tobacco member (NtPSY3) was recovered in subgroup E3 in our study (**Figure 2**), whereas two members NtPSY3–1 and NtPSY3–2 were reported previously (Wang et al., 2021). This discrepancy likely reflects the loss of one PSY3 homoeolog in the current reference due to pseudogenization and/or assembly collapse in the latest genome build. Moreover, neither NtPSY3–1 nor NtPSY3–2 showed detectable transcripts in any of the four tissues assayed previously (Wang et al., 2021), further suggesting that at least one *PSY3* copy may represent a pseudogene or a low-confidence locus. Notably, *NtPSY3* was found to be expressed in flowers in our study (**Figure 6**), hinting that it may have a role in flower development.

Phytoene synthase has been reported to control the synthesis of carotenoids and flavonoids in plants (Cao et al., 2024; Rodríguez-Villalón et al., 2009). Notably, SlPSY1 from subgroup E1 was characterized as a specific carotenoid regulator in tomato. SlPSY1 controls carotenoid biosynthesis mainly in the leaf and fruit, respectively (Cao et al., 2024). The evolutionary analysis showed that NtPSY1, NtPSY2, NtPSY4, and NtPSY5 were clustered together within subgroup E1 (**Figure 2**). Furthermore, the transcriptome data showed that these tobacco *PSY* genes were highly expressed in the leaf (**Figure 6**), hinting that these genes might control carotenoid biosynthesis in tobacco leaves. Besides that, many phytoene synthase family members were found to confer tolerance to abiotic stresses in plants (Cidade et al., 2012; Han et al., 2008). In subgroup E1, *NtPSY1* was clustered with *AtPSY1* and *SlPSY1* (**Figure 2**). *SlPSY1* had been reported to be induced by cold stress. Interestingly, the promoter analyses revealed that the promoter region of *NtPSY1* gene contains many stress response *cis*-elements (**Figure 5**), suggesting that it might be involved in stress response. Furthermore, *NtPSY1* genes were detected to be induced by cold treatment (**Figure 7**, **Supplementary Figure 1**), implying that *NtPSY1* might be involved in cold stress responses in tobacco. In addition, the overexpression analyses further demonstrated that *NtPSY1* can confer carotenoid accumulation and cold tolerance in transgenic tobacco plants (**Figure 8**). Those clues indicated that *NtPSY1* acts as an activator to regulate carotenoid accumulation and cold stresses. Notably, upon *NtPSY1* overexpression, the transcript levels of *NtCCD7*, *NtCCD8*, and *NtMAX1* increased (**Supplementary Figure 3**), consistent with enhanced carotenoid precursor supply and activation of the strigolactone biosynthetic pathway. This interpretation aligns with the established role of PSY in controlling carotenoid flux toward SL biosynthesis.

## 5 Conclusions

The systematic analysis of the tobacco genome sequences in this study was carried out to identify and characterize the phytoene-synthase-encoding genes. The phylogeny and the expression profiling analysis implied that the tobacco *PSY* gene family might be involved in various biological processes. The PSY members homologous within *Solanaceae* were found to play conserved roles in regulating plant development and stress responses. Notably, NtPSY1 was induced by cold treatments. Furthermore, the overexpression of *PSY1* in tobacco significantly enhanced carotenoid accumulation, strigolactone biosynthesis, and the cold stress tolerance of the transgenic plants.

## Data Availability

The original contributions presented in the study are included in the article/**Supplementary Material**. Further inquiries can be directed to the corresponding authors.

## Supplementary material

Supplementary Table 1 Detailed information on the qRT-PCR primers used in this study.

Supplementary Table 2 Detailed information on the identified NtPSY members.

Supplementary Table 3 Synteny blocks of *NtPSY* genes between tobacco and other plant species.

Supplementary Table 4 RNA-seq raw data of *NtPSY* genes from the tested tissues.

Supplementary Table 5 CDS and protein sequences of all NtPSY members.

Supplementary Figure 1 Conserved exon–intron structures of NtPSY members.

Supplementary Figure 2 qRT-PCR analysis of representative *NtPSY* genes under cold stress.

Supplementary Figure 3 Expression level of *NtPSY1*, *NtCCD7*, *NtCCD8*, and *NtMAX1* genes in wild type and two overexpression lines.
