# Supplementary figures and images for: Diversification of tobacco phytoene-synthase-encoding genes coordinates carotenoid flux, strigolactone biosynthesis, and cold responses
Source: Front Plant Sci. 2026 Apr 10;17:1750572. doi: 10.3389/fpls.2026.1750572 (PMC13106284; doi:10.3389/fpls.2026.1750572)

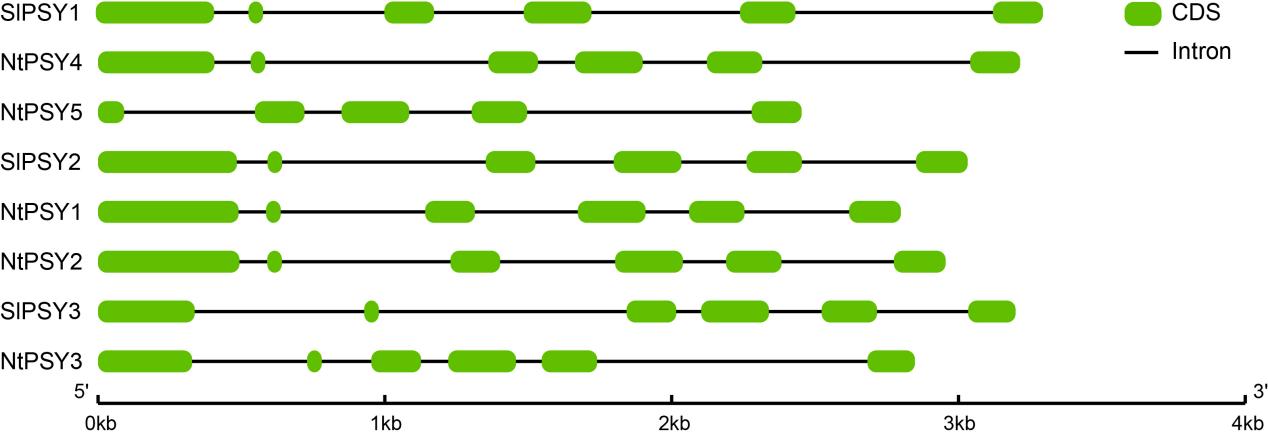

Supplement: Supplementary Table 1 — Detailed information on the qRT-PCR primers used in this study. [file DataSheet1.zip › Supplementary files/Supplementary Figure S1. Conserved exon-intron structure organizations of NtPSY members.jpg]

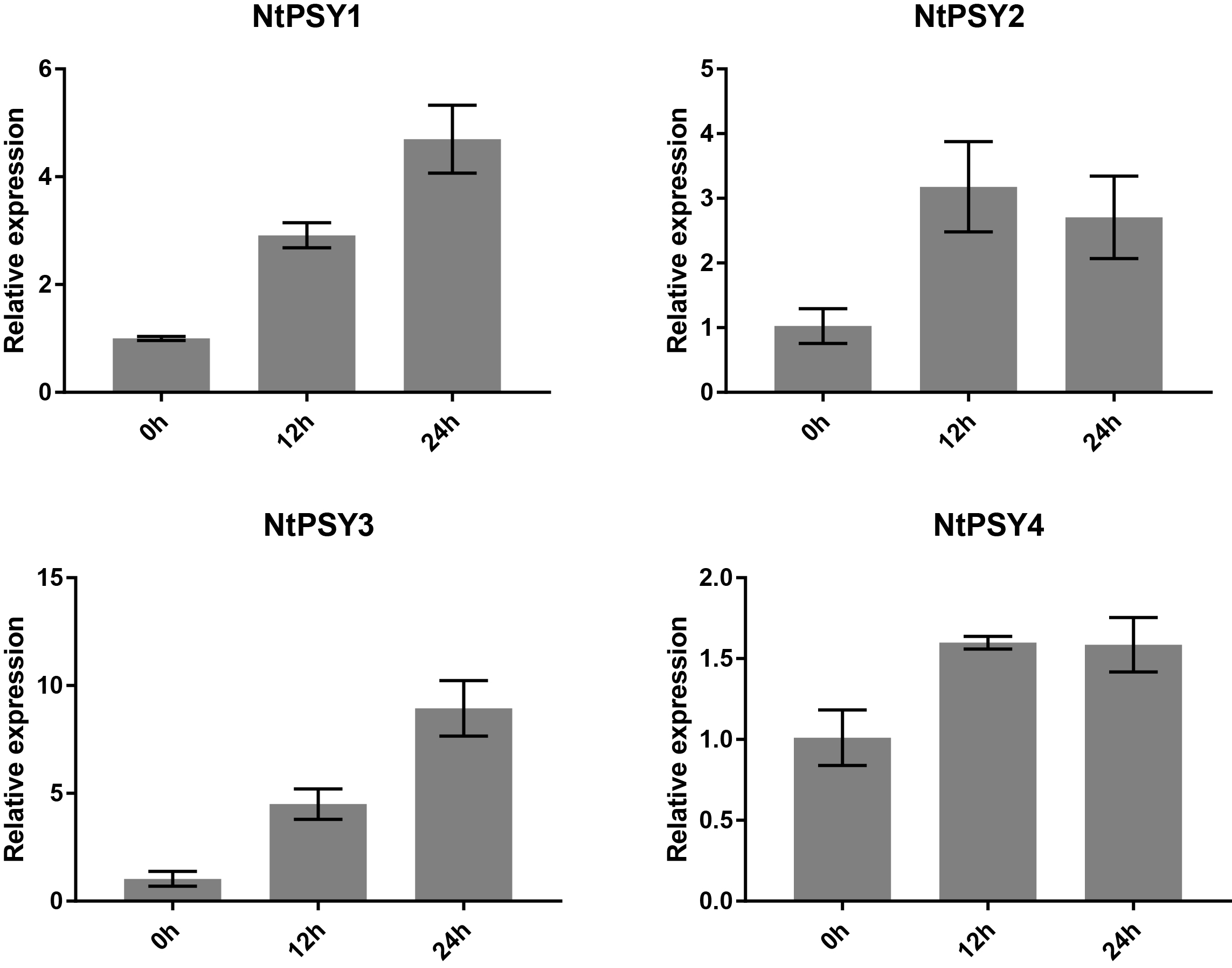

Supplement: Supplementary Table 1 — Detailed information on the qRT-PCR primers used in this study. [file DataSheet1.zip › Supplementary files/Supplementary Figure S2. qRT-PCR analysis of representative NtPSY genes under cold stress. .jpg]

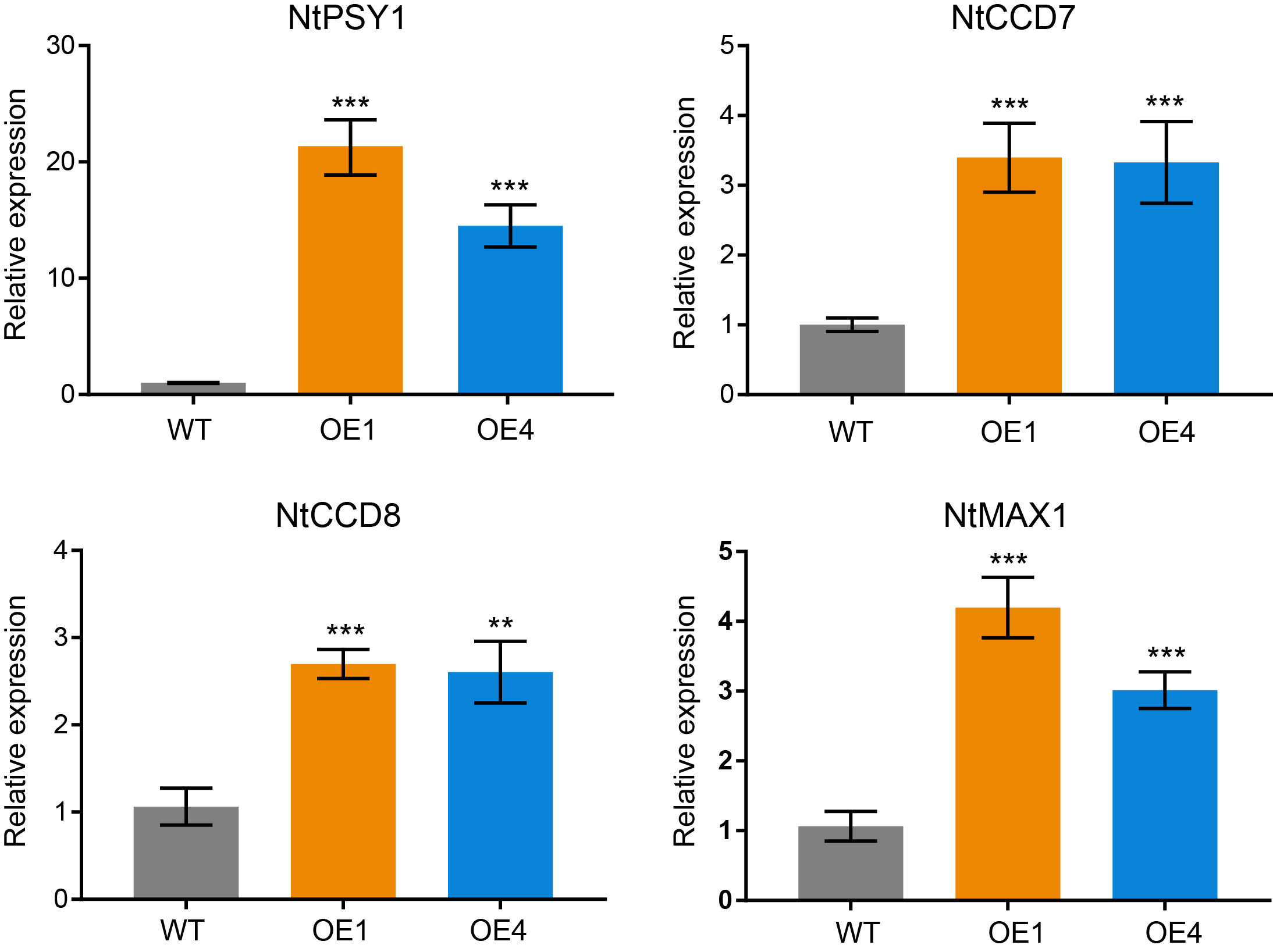

Supplement: Supplementary Table 1 — Detailed information on the qRT-PCR primers used in this study. [file DataSheet1.zip › Supplementary files/Supplementary Figure S3. The expression level of NtPSY1, NtCCD7, NtCCD8 and NtMAX1 genes in wild type and two overexpression lines.jpg]
